# Assessing the Economic Feasibility of Assuring Nutritionally Adequate Diets for Vulnerable Populations in Uttar Pradesh, India: Findings from a “Cost of the Diet” Analysis

**DOI:** 10.1093/cdn/nzaa169

**Published:** 2020-11-13

**Authors:** Shivani Kachwaha, Phuong Hong Nguyen, Michelle DeFreese, Rasmi Avula, Shruthi Cyriac, Amy Girard, Purnima Menon

**Affiliations:** International Food Policy Research Institute, Washington, DC, USA; International Food Policy Research Institute, Washington, DC, USA; Congressional Hunger Center, Washington, DC, USA; International Food Policy Research Institute, Washington, DC, USA; Hubert Department of Global Health, Emory University, Atlanta, GA, USA; Hubert Department of Global Health, Emory University, Atlanta, GA, USA; International Food Policy Research Institute, Washington, DC, USA

**Keywords:** cost of diet, affordability, accessibility, India, nutritious diet

## Abstract

**Background:**

Healthy diets can help reduce undernutrition, morbidity, and mortality. However, evidence on the accessibility and affordability of recommended diets is limited, particularly in poor-resource settings including India.

**Objectives:**

This study examined: *1*) the minimum cost of different types of household diets; *2*) how economic constraints can prevent households from accessing a nutritious diet; and *3*) how home production and social protection can improve access to nutritious diets.

**Methods:**

We conducted 24 market and 125 household surveys in Uttar Pradesh, India, to obtain food prices and consumption patterns. Cost of Diet, a linear programming software, was used to assess the minimum cost of different diets, estimate affordability of nutritious diets, and model scenarios of home production and social protection interventions to improve affordability.

**Results:**

The minimum-cost nutritious diet that met all recommended nutrient requirements [904 US dollars (US$)/y] was over twice as expensive as the diet that only met energy requirements (US$393/y). The nutritious diet was unaffordable for 75% of households given current income levels, consumption patterns, and food prices. Household income and dietary preferences, rather than food availability, were the key barriers to obtain nutritious diets. Home production had potential to reduce the cost of nutritious diets by 35%, subsidized grains by 19%, and supplementary food by 10%. The poorest households could only afford recommended nutritious diets with access to multiple interventions.

**Conclusions:**

Practical, habitual, diet-related behavior change communication to middle- and high-income households and additional social protection for poorer households could enable individuals to achieve optimal nutrient intakes.

## Introduction

Poor dietary intake remains a significant public health concern with several adverse consequences. These consequences especially impact pregnant women and children, and include maternal anemia (1), preterm delivery (2, 3), small- and large-for-gestational age babies (4–6), and impaired child cognitive and behavioral outcomes (7). Inadequate and imbalanced diets are responsible for substantial morbidity and mortality, with an estimated 11 million deaths and 255 million disability-adjusted life years being attributable to dietary risk factors across 195 countries in 2017 (8).

Current trends in global dietary patterns indicate a suboptimal consumption of healthy foods and excessive consumption of unhealthy foods. Whereas mean consumption of nuts and seeds, milk, and whole grains is low at 12–23% of the optimal intake, consumption of sugar-sweetened beverages, processed meats, and sodium is 90% higher than the optimal intake on average (8). In low- and middle-income countries, dietary patterns are often heavily cereal based with imbalanced consumption of macronutrients and inadequate consumption of micronutrients (9). Key factors associated with poor dietary intake are food insecurity, economic constraints, limited knowledge of nutritious diets, and food preferences (10, 11).

India has one of the highest burdens of maternal and child malnutrition globally, with nearly one-third of women having low BMI (<18.5 kg/m^2^), 38% of children being stunted, and more than half of pregnant women and children being anemic (12). Along with persisting undernutrition, overweight and obesity is a growing concern, with nearly 1 in 5 women having a BMI ≥25 (12). Suboptimal diets have been identified as the key contributing factor for malnutrition in the “nutrition transition” period over the last 30 y, characterized by a decreased consumption of coarse cereals, pulses, fruits, and vegetables, and an increased consumption of salt and unhealthy foods (13).

Alive and Thrive (A&T) is an initiative to save lives, prevent illness, and ensure healthy growth and development through optimal maternal nutrition, breastfeeding, and complementary feeding practices (14). A&T is implementing a maternal nutrition intervention in Uttar Pradesh, India, aiming to improve the provision and uptake of a package of maternal nutrition services, with improving dietary intakes for pregnant women being the most important component of the intervention, specifically counseling on nutritious diets and recommended foods during pregnancy. However, the efficacy of such behavior change communication and related strategies is dependent on the availability, accessibility, and affordability of recommended dietary practices as well as food habits of the local population. Previous literature in some developing countries has shown that income levels, food variety in local markets, and food habits are important determinants of consuming nutritious diets (15–21), but evidence of costs and preferences associated with recommended diets of pregnant women is limited, particularly in India.

Cost of the Diet (CotD) is an assessment tool developed by Save the Children that uses linear optimization to estimate the amount and a combination of local foods that are needed to meet the average needs for energy, protein, fat, and micronutrient requirements for a typical household (22). The tool is designed for informing policy decision-making, and program design of effective interventions by selecting the most nutritious and least expensive foods to meet energy and nutrient specifications using local foods. CotD has been widely used and found to be valid and reliable in estimating locally available and cost-effective nutritious diets in several countries, including Bangladesh, Indonesia, Pakistan, and Kenya (15, 17, 19, 23).

We integrated a CotD assessment within the overall impact evaluation of the A&T maternal nutrition intervention in Uttar Pradesh to: *1*) estimate the minimum cost for different types of diets for typical households and pregnant women in Uttar Pradesh; *2*) examine the extent to which economic constraints might prevent households from having access to a nutritious diet; and *3*) assess how home production and social protection strategies might contribute to improved access to nutritious diets.

## Methods

### CotD assessment tool

The CotD software comprises 5 built-in databases: *1*) food composition tables with data on energy and nutrient content of foods (22); *2*) the energy and nutrient requirements of individuals based on WHO standards; *3*) typical household compositions; *4*) portion sizes of foods; and *5*) currency conversion factors. A typical CotD assessment includes 3 types of complementary data collection: *1*) a market survey to obtain price data of available food items; *2*) a household survey to capture typical food habits; and *3*) a secondary data source to identify wealth groups.

### Study design and data collection

This assessment followed the overall cluster-randomized controlled design of the maternal nutrition intervention evaluation study. Among the 26 rural blocks from Unnao and Kanpur Dehat districts in Uttar Pradesh, 12 were randomly selected covering both treatment and control areas. Within each block, 2 Gram Panchayats (GPs) were randomly selected from the baseline sample for a total of 24 GPs. In each GP, a commonly visited market area was identified based on local informants for market survey. In addition, 5–6 pregnant women were randomly sampled from the list of pregnant women for the household survey. The total sample included 24 market areas and 125 households with pregnant women. Information on details of data collection for price data, food habits, and income for wealth groups are presented in the subsequent sections.

#### Market survey to collect price data

A list of all food items available in the markets was developed using 3 sources: data from 24-h dietary recall in the baseline survey 2017 (24), consultations with local key informants, and field testing in 9 local markets. In total, 158 food items were included in the food list, among them 137 food items were available in the local markets at the time of study (**Supplemental Table 1**).

Price and weight data for every available food item in the food list were collected from 1–3 randomly chosen vendors in each market area. Market vendors were first asked the price of the smallest unit they usually sell for each food in the current (winter) season. Samples of food items were then weighed 1–3 times using electronic scales with 1-g precision. Thus, a possible total of 72 price points and 216 weights for each food item were collected in 24 markets.

#### Household food habits survey

We interviewed members responsible for cooking in the households of pregnant women to understand food habits and frequency of consumption for each item in the food list. The defined frequencies were: never (0 times), rarely (<1 time/wk), often (1–4 times/wk), and usually (>5 times/wk). Respondents were also asked about the main sources of foods: purchased from market, produced at home, or wild/foraged foods obtained for free.

Data were collected using a structured questionnaire administered on tablets (household) and paper form (market) by the survey team appointed by Network for Engineering and Economics Research and Management. Survey enumerators were trained through lectures, role play, mock interviews in classroom settings, and during field practice. Ethical approval was obtained from Suraksha Independent Ethics Committee in India and the International Food Policy Research Institute in the United States. Verbal informed consent was obtained from all participants. Data collection took place in November 2018.

#### Assessment of income for different wealth groups

Incomes for different wealth groups were obtained from the National Sample Survey Office consumer expenditure data 2011–12 (25). This survey uses monthly per capita consumer expenditure, including both food and nonfood expenditure items, to measure standard of living. Inflation was adjusted for using the Consumer Price Index (CPI) by the inflation-adjusted formula: [{CPI 2018 × Income 2012(14)}/CPI 2012(14)]. Disaggregated data for rural Uttar Pradesh were available from 5916 households across 740 villages. The major nonfood expenditure items included fuel, electricity, medical expenses, transportation, and clothing (25). A wealth index was calculated using monthly per capita consumer expenditure for household annual income and nonfood expenditure, and was then categorized into quartiles to define the wealth groups (very poor, poor, middle, and better-off). The household annual income ranged from 50,919 to 211,792 Indian rupees (INR) [717–2981 US dollars (US$)], and nonfood expenditure ranged from INR 20,052 to 126,172 (US$282–1776).

### Data analyses

The key steps of data analyses are presented in the sections below: *1*) estimating minimum cost for 4 types of diet; *2*) examining nutrient requirements met in each diet; *3*) estimating affordability of each diet and modeling scenarios of interventions to improve affordability; and *4*) identifying affordable and accessible foods that meet the majority of nutrient requirements in the diets. Treatment and control areas were pooled together for the analysis because we did not expect the maternal nutrition intervention to influence food prices.

#### Estimation of minimum cost of diet

The market price, weight, and food consumption data were entered in the CotD software. An average price per 100 g was generated for 137 food items found across all market areas (Supplemental Table 1). A food habits score (range 0–16) was constructed based on average frequency of consumption for each food. A household of 6 members was defined for rural Uttar Pradesh using baseline survey data (24) from the impact evaluation and included: a grandmother, a pregnant woman with her husband, and 3 children (1 of which is aged <24 mo and breastfeeding).

Based on market prices, food habits, family composition, and nutrient requirements, 4 theoretical diets were generated: *1*) a lowest cost diet that only meets recommended average energy requirements (energy-only diet); *2*) a lowest cost diet that only meets the average energy and the recommended protein and fat requirements (macronutrients diet); *3*) a lowest cost diet that meets recommended intakes for energy, protein, fat, and 13 micronutrients (nutritious diet); and *4*) a lowest cost diet that reflects typical dietary habits in addition to meeting recommended nutrient intakes (food habits-based nutritious diet). The average annual cost of each diet is given in Indian rupees and US dollars using an exchange rate of INR 71.04 per US dollar (26). These diets were estimated separately for the household overall and for pregnant women within the household.

#### Estimation of the extent to which nutrient intake requirements were met in the diets

Each of the 4 diets generated indicate the extent to which nutrient requirements were met, including energy, protein, fat, and 13 micronutrients. The individual nutrient specifications are embedded in the software, specifying the estimated average requirement and recommended dietary allowance by WHO and FAO (22), which are compatible with the estimated requirements by the Indian National Institute of Nutrition, except for lower folic acid and higher iron (27) (**Supplemental Table 2**). Although individual portion sizes and nutrient intakes are specified, the software does not factor intrahousehold distribution of resources (28).

The specification for protein intake is based on a database that uses the 95th percentile recommended by FAO and WHO. Individual fat needs are specified between 30% and 60% of total energy intakes. Energy intakes are set not to exceed 100% of requirements in any diet. Micronutrient needs are taken from a database that specifies the 97.5th percentile of recommended nutrient intake by WHO and FAO. In addition, absorption factors are applied for each food item in the database to take into account the bioavailability of nutrients from the diet (28). We compared the 4 diets to estimate the extent to which recommended nutrient intakes were met.

#### Estimate the affordability of diets

Affordability of each type of diet for households in different wealth groups was estimated by comparing the cost of different diets plus nonfood expenditure with correspondent total income. These costs are presented both as absolute numbers and as percentage of annual income.

In addition, we modeled the potential influence of home production and social protection on the affordability of diets. Based on the responses of the household survey, we identified 7 key food items that were home produced by >50% of households, including wheat, maize, buffalo milk, cow milk, mustard spinach, chickpea leaf, and goosefoot leaf. We then estimated a weighted average price of the proportion home producing [price adjusted to 20% of market cost to account for cost of production (28)] and the proportion not home producing (price unchanged) different foods to model the influence on cost of diet and affordability.

For social protection, we modeled both food-based transfers and cash transfers. The Integrated Child Development Services provides take-home rations (THRs) for children aged 6 mo to 3 y and for pregnant women. The THR is a packaged dry mix consisting of wheat flour, soya flour, maize, rice, sugar, and fat. To model the potential influence on diet cost and affordability, we entered THR as a new food in the software (**Supplemental Table 3**) and included 125 g of free THR with a maximum constraint of 21 servings in a month as per government norms (29).

We also modeled the influence of subsidized grains provided by the government's Public Distribution System (PDS) by cloning wheat flour from the database as a separate food with price INR 2/kg (price of subsidized wheat was found to be the same in ration shops for all households during fieldwork). In addition, we examined the potential influence of the national cash transfer scheme for pregnant women on diet affordability by adding an amount of INR 5000 to household annual income (30). Finally, we modeled a scenario of home production and social protection together to estimate the combined influence of different interventions on the affordability of diets.

#### Identification of inexpensive and nutrient-rich local foods that could be promoted through maternal nutrition interventions

The lowest cost nutritious diets generated potentially indicate nutrient-rich local foods that could be promoted by interventions. We examined the compositions of the nutritious diet and the food-habits nutritious diet to identify foods that are inexpensive, accessible in local markets, rich in essential nutrients, and acceptable. Within each diet, we selected 5–6 foods, each of which provide the majority of requirements for ≥1 macronutrients or micronutrients in that diet.

## Results

### The availability of foods in the local markets

A total of 137 foods items were available in the markets at the time of assessment (Supplemental Table 1). They included 12 types of grains, 6 types of white roots and tubers, 14 types of pulses, 6 types of nuts and seeds, 6 types of dairy products, 17 types of meat and fish, 33 types of vegetables, 18 types of fruits, 5 types of oils and fats, 4 types of sweets and snacks, and 16 types of condiments. The availability of different food items varied considerably across markets. The most commonly available food items (found in >20 markets) were wheat, rice, potato, lentils, beans, milk, several vegetables and fruits, and eggs. Less commonly available foods found in <10 markets included millets, white-fleshed sweet potato, parts of chicken and goat, fish, and nuts and dry fruits.

### Typical food consumption habits

Frequency of consumption based on the food habits score (range 0–16) also varied distinctly by types of food (Supplemental Table 1). The most commonly consumed items (score 14–16) consisted primarily of grains, sugars, fats, condiments, and beverages. This group included foods such as wheat, rice, sugar, oil, onion, tomato, potato, spices, and tea. Foods that were least commonly consumed (score 0–2) included all types of animal source foods (meat, fish, and eggs), nuts (walnut and pistachio), and dry fruits (figs).

### Cost and composition of diets

The average annual cost for a household of 6 members varied considerably by types of diet, ranging from INR 27,892 (US$393) for the energy-only diet, INR 29,254 (US$412) for the macronutrient diet, INR 43,128 (US$607) for the nutritious diet, and INR 64,225 (US$904) for the food-habits nutritious diet (**Table 1**). The cost of the food-habits nutritious diet was therefore nearly 2.5 times the cost of the energy-only diet. The cost of diet for pregnant women accounted for ∼25–30% of total cost of the household diet.

**TABLE 1:** Diet composition and cost, by type of diet for household and pregnant woman^1^

Type of diet	No. of foods	Food groups	Annual cost (INR)	Annual cost (US$)
Household				
Energy-only diet	3	Cereals, dairy^2^	27,892	393
Macronutrient diet	4	Cereals, dairy,^2^ fat	29,254	412
Nutritious diet	12	Cereals, dairy,^2^ legumes, vegetables, meat, fish, sugar, fat	43,128	607
Food habits-based nutritious diet	22	Cereals, dairy, legumes, vegetables, eggs, fruit, sugar, fat	64,225	904
Pregnant woman				
Energy-only diet	2	Cereals	7103	100
Macronutrient diet	3	Cereals, fat	7430	105
Nutritious diet	7	Cereals, meat, vegetables, fat	10,764	152
Food habits-based nutritious diet	14	Cereals, dairy, legumes, vegetables, eggs, fruit	19,273	271

1 INR, Indian rupees; US$, US dollars.

2 Only breastmilk and no other dairy sources are included in these diets.

The composition of the 4 diets differed substantially (Table 1). The energy-only diet was the least diverse diet, consisting of only 2 food groups (cereals, breastmilk). The energy-only diet met 100% of energy requirements, but not those for fat (28%) or several micronutrients including vitamins A, C, and B-12 (<10%), calcium (23%), folic acid (50%), and iron (76%) (**Table 2**). The macronutrient diet had a similar composition to the energy-only diet but with the addition of fat, therefore the nutrients met by the macronutrient diet were nearly identical to energy-only, with the addition of 100% of fat requirements met. In contrast, the nutritious and food-habits nutritious diets were considerably more diverse, comprising 8 food groups (12–22 food items); thus these 2 diets met or exceeded requirements for all macro and micronutrients. Detailed information on food items included in each diet and their nutrient values are given in **Supplemental Tables 4–7**.

**TABLE 2:** Nutrient requirements met, by type of diet

	Percentage of nutrient requirements met			
Nutrient	Energy-only diet	Macronutrient diet	Nutritious diet	Food habits-based nutritious diet
Energy	100.0	100.0	100.0	100.0
Protein	220.9	188.3	219.9	235.1
Fat	28.0	100.0	100.0	102.2
Vitamin A	8.2	8.2	361.3	145.6
Vitamin C	9.1	9.1	227.3	101.6
Vitamin B-1	194.2	169.3	169.9	183.6
Vitamin B-2	101.5	89.2	136.9	191.9
Niacin	389.0	338.5	345.9	313.7
Pantothenic acid	118.6	103.0	112.0	128.0
Vitamin B-6	137.5	120.5	161.1	151.3
Folic acid	50.6	43.7	119.1	126.1
Vitamin B-12	5.0	5.0	111.0	100.0
Calcium	22.7	20.1	100.0	105.5
Iron	76.1	66.3	147.5	100.0
Magnesium	443.0	388.8	655.2	451.8
Zinc	252.2	220.8	260.2	252.8

### Affordability of different diets

**FIGURE 1:**
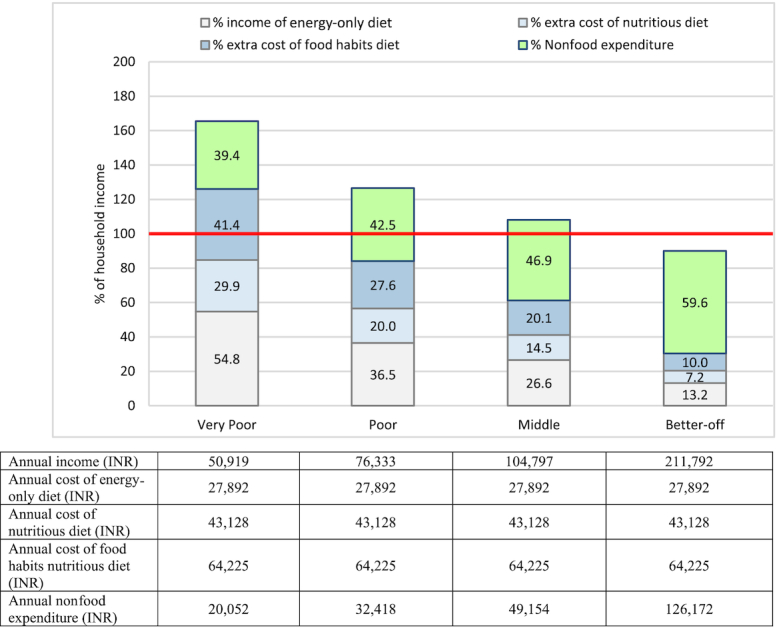
Affordability of diets by wealth group. Income available for food = annual income − annual nonfood expenditure. US$1 ∼ INR 71.04. INR, Indian rupees; US$, US dollars.

The relative affordability of each diet as a proportion of household income varied for different wealth groups (**Figure 1**). Given current income levels and minimum diet cost, very poor households would need to spend over half their income just to afford the energy-only diet. These households would require nearly 85% of their income to obtain the nutritious diet (54.8% for energy-only plus 29.9% extra for nutritious), which is unaffordable when essential nonfood expenditure is considered (additional 39.4%). Poor and middle households can barely afford the nutritious diet in addition to meeting their nonfood expenditure. The food-habits nutritious diet is unaffordable for the majority of households except the better-off group.

### Influence of home production and social protection on affordability of diet

Home production, which was estimated at 20% of market cost for 7 common food items (wheat, maize, buffalo milk, cow milk, mustard spinach, chickpea leaf, and goosefoot leaf) among half of the sample, had the largest potential effect on reducing the cost of diet. The cost of the food-habits nutritious diet reduced from INR 64,225 (US$904: standard diet cost) to INR 41,186 (US$580) with home production, a reduction of 35%, thus enabling poor and middle households to afford the diet. Access to subsidized wheat through PDS reduced diet cost by a relatively lesser degree (INR 51,813 or US$729, reduction of 19% from standard cost), bringing the food-habits nutritious diet within the reach of middle-income households.

In-kind food transfers (THRs) had a small relative effect on improving affordability. In the scenario of THRs being available for children aged <3 y and pregnant women, the diet cost would reduce by ∼10% (INR 57,595 or US$811 from standard cost). This would allow middle-income households to afford the food-habits nutritious diet only if a cash transfer amount of INR 5000 (US$70) was available along with THRs. Therefore, neither cash transfers nor THRs had considerable individual influence on improving diet affordability for any wealth group, unless simultaneously available.

**FIGURE 2:**
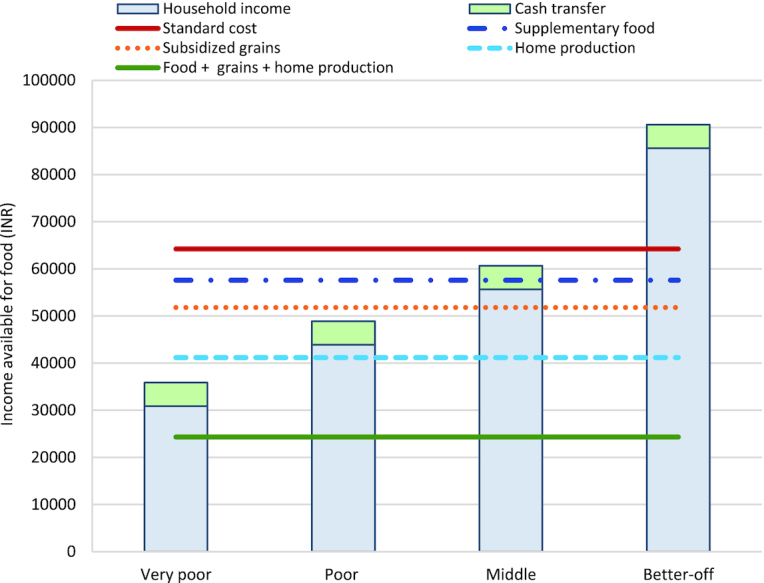
Influence of home production and social protection on affordability of food habits-based nutritious diet, by wealth group. Subsidized grains refers to wheat distributed through the Public Distribution System. Supplementary foods include take-home rations provided through the Integrated Child Development Services. Home production includes 7 common foods items: wheat, maize, buffalo milk, cow milk, mustard spinach, chickpea leaf, and goosefoot leaf. US$1 ∼ INR 71.04. INR, Indian rupees; US$, US dollars.

The greatest potential influence on improving affordability of the food-habits nutritious diet is a scenario of combined home production and social protection. If households received THRs, PDS, and home-produced certain food items, diet cost reduced by nearly 60% (INR 24,338 or US$343 from standard cost), thus enabling all households to obtain the food-habits nutritious diet (**Figure 2**). The poorest wealth quartile would only be able to afford the food-habits nutritious diet in the scenario of combined availability of home production and access to social protection schemes.

### Inexpensive local foods that could provide essential nutrients and be promoted through program interventions

We identified key affordable nutrient-dense foods within the nutritious and food-habits nutritious diets for promotion through existing program interventions (**Table 3**). The identified foods were locally available in the market areas, contained a majority of macro or micronutrients in the diets, and were the least expensive compared with alternative foods of similar nutrient composition. In the nutritious diet, chicken liver and minnow fish provided the majority of vitamin B-12 requirements (80% and 16%, respectively), amaranth leaf provided substantial vitamins A (82%) and C (95%), folic acid (45%), calcium (62%), and iron (35%), and vegetable oil provided the majority of fat requirements (65%).

**TABLE 3:** Inexpensive sources of essential nutrients, by type of diet

	Percentage of nutrient requirements met										
	Nutritious diet						Food habits-based nutritious diet				
Nutrient	Chicken liver	Minnow fish	Amaranth leaf	Oil	Wheat flour	Egg	Mustard spinach	Buffalo milk	Soya bean	Spinach	Wheat flour
Energy	0.6	0.6	3.5	13.6	59.6	1.7	0.1	15.9	3.2	1.2	40.7
Protein	2.2	2.3	13.2	0.0	63.9	3.5	0.3	16.3	6.6	3.9	40.8
Fat	1.0	1.0	1.5	64.6	13.7	4.7	0.1	43.1	5.8	0.6	9.1
Vitamin A	15.4	0.0	82.1	0.0	0.0	4.9	5.7	23.0	0.0	50.4	0.0
Vitamin C	0.0	0.0	95.7	0.0	0.0	0.0	29.6	7.5	2.0	24.4	0.0
Vitamin B-1	1.9	0.6	4.0	0.0	78.0	0.9	0.4	9.2	7.2	6.0	49.3
Vitamin B-2	13.2	0.8	21.4	0.0	50.1	6.0	0.5	29.2	6.7	13.5	24.4
Niacin	2.1	0.0	8.3	0.0	76.5	1.5	0.2	8.9	0.6	3.2	57.7
Vitamin B-6	4.6	1.7	23.0	0.0	59.6	1.5	0.9	9.3	3.2	14.7	43.3
Folic acid	14.3	0.0	45.7	0.0	26.7	2.3	3.6	4.5	12.6	35.5	17.2
Vitamin B-12	79.8	15.7	0.0	0.0	0.0	13.1	0.0	78.8	0.0	0.0	0.0
Calcium	0.2	3.8	62.7	0.0	13.4	1.2	2.2	50.7	4.3	15.1	8.7
Iron	11.6	6.7	34.2	0.0	35.6	5.1	0.6	0.0	8.7	14.5	35.9

In the food-habits nutritious diet, mustard spinach provided considerable vitamin C (∼30%) and buffalo milk provided the majority of fat (43%), vitamins B-2 (29%) and B-12 (78%), and calcium (50.7%). Spinach provided considerable vitamin A (50%), iron (15%), and folic acid (35%). Wheat flour provided the bulk of remaining macro and micronutrients including energy, protein, vitamins B-1, B-2, B-6, and niacin in both the nutritious and food-habits nutritious diets.

## Discussion

Our study applied the cost-of-diet (CotD) approach to the Indian context to fill critical gaps in knowledge related to the affordability, availability, and accessibility of nutritious diets. We found that the energy-only and macronutrient diets were affordable for all households (US$393/y and US$412/y, respectively), but did not meet requirements for several key nutrients. The nutritious diet (US$607/y) and food habits-based nutritious diet (US$904/y) met nutrient requirements but were unattainable for most households except the better off. Home production and social protection strategies including in-kind food transfers, food subsidies, and cash transfers could play an important role in reducing the affordability gap for obtaining the food habits-based nutritious diet.

Compared with previous CotD assessments in other South Asian contexts (17, 18, 20, 21), our assessment area yielded relatively more varieties of foods, which should be sufficient to meet nutrient requirements for a typical household. Although food items were abundantly available, households mainly consumed grains, starchy staples, and sugar. The most frequently consumed items included wheat, rice, potato, and oil, and least frequently consumed items included fish, meat, and nuts. This is consistent with previous dietary assessments highlighting that diets of pregnant women in rural Uttar Pradesh are heavily dominated by grains, with low consumption of animal-source foods, fruits, and vegetables (11). Thus, economic constraints and dietary habits rather than food availability were the key barriers for poor households to obtain a nutritious diet.

The optimal food habits-based nutritious diet was typically 2.5–3 times costlier to obtain than the energy-only diets (17, 18, 20, 21). Consistent with global trends, the food-habits diet in our assessment was substantially more expensive and was well beyond the reach of very poor, poor, and even middle-wealth group households. This resonates with the idea that food budgets in poverty are usually insufficient to ensure optimum diets (10). For example, national survey data from India show that on average, households spend ∼US$554 annually on foods, whereas the food habits-based nutritious diet in our assessment costs nearly double at US$930 (25). At the individual level, we estimate that 25–30% of household food expenditure would need to be allocated for pregnant women to obtain the optimum diet. However, general patterns of intrahousehold food allocation highlight inequitable distribution disadvantaging women, particularly in situations of food insecurity (31). These findings point to the significant barriers of accessing nutritious diets in the context of high food prices relative to low household income, food insecurity, and inequity.

When estimating the potential individual impact of home production or different social protection interventions to improve the affordability of minimum-cost nutritious diets, we found home production to be associated with the largest potential reduction in diet cost (35%), followed by subsidized grains (19%), and THRs (10%). Thus, home production of 7 common food items alone could potentially enable poor and middle-income households to obtain the food habits-based nutritious diet, whereas subsidized wheat alone could enable only middle-income households to afford that diet. Neither THRs nor cash transfers alone had individual impacts on affordability, but combination of these 2 interventions enabled middle-income households to obtain the optimal diet.

Evidence from 10 South Asian and African assessments document a 10–30% potential improvement in affordability for individual interventions in different contexts, with food transfers being marginally more effective in improving optimal diet affordability compared with cash transfers (15–21, 23, 32, 33). However, the effectiveness of food compared with cash transfers is likely to be driven by complex context-specific factors, including the composition of food supplements and the amount of cash relative to food prices and household income. In our context, we found that food transfers through subsidized grains (PDS) were generally associated with larger potential reductions in cost, but supplementary food (THRs) was associated with the smallest individual reduction. This could be likely be due to several factors, including that THRs are provided only for targeted members within the household, that competing low-cost foods that could replace nutrients provided by THRs are widely available, or that components of THRs are relatively low cost in market areas.

Previous CotD research showed that the poorest wealth groups could not afford the food-habits nutritious diet through the modeling of any single intervention (15–21). Therefore, we estimated models of multiple home production and social protection interventions together. We found that combination of these interventions resulted in a potential 60% reduction in diet cost, so that even the very poor households could obtain the optimal diet. Although promising, this hypothetical “optimal scenario” of access to multiple interventions together is likely to have several constraints, including significant barriers in coverage and utilization of subsidized wheat through PDS and supplementary food through THRs. Previous studies on PDS highlighted several issues in its functioning including poor targeting of beneficiaries, exclusion of the poorest households, corruption, supply shortages, and low rates of purchase by intended beneficiaries (34, 35). Supplementary food has been associated with low receipt among beneficiaries, irregularities in supply, quality concerns about rations, leakage and targeting issues, and lack of awareness about entitlements (36–38). Similarly, home production is likely to have several land- and resource-related constraints, whereas cash transfers entail considerations of financial literacy, banking coverage, and market stability for their effective delivery (39).

We acknowledge some methodological limitations of our analyses. First, data were available for the winter season only, so we could not consider seasonal variations in food prices and availability. During field testing, we found that retrospective recall of prices from previous seasons imposed a considerable cognitive burden on participants, thereby leading to unreliable price estimates. Second, although the CotD software allocates specific types and amount of foods within the household diet to individual members, we do not know how the dynamics of intrahousehold allocation operate in reality. Lastly, our modeling of home production and social protection interventions has some caveats. Although some of the interventions (such as THRs and cash transfers) are targeted to specific individuals, we modeled them at the household level because the income estimates were available for the household only. For home production, we are not able to account for the opportunity costs for households to produce specific crops. Our modeling was based on the optimal impact of 7 foods that the majority of households in our sample stated were home produced. However, individual households might be producing fewer foods, which could lead to overestimation of the impacts seen in our model. We use the overall assumption of estimating home production at 20% of market costs rather than using specific agricultural production costs. Therefore, it is possible our estimates could either over- or underestimate true production costs.

Our study provides unique insight into the costs and economic constraints to obtain nutritious diets in the context of a large-scale maternal nutrition intervention in rural Uttar Pradesh. Although several nutrition interventions aiming to improve dietary intakes through strategies related to behavior change communication, agricultural activities, or food fortification have shown moderate impacts (40–44), information related to factors associated with recommended diets (such as accessibility, affordability of recommended dietary practices, or food habits of the local population) is lacking. Our study bridges the gaps in the literature by using the CotD method to estimate the minimum cost and affordability of 4 different types of diets. Using innovative modeling, we provide new information on how optimal diets can be made more affordable for poor households through the CotD software that considers absorption factors for bioavailability and uses WHO nutrient recommendations, which are similar to Indian guidelines. We estimate the potential impact of 4 policy-relevant interventions both individually and in combination, finding that the poorest households can only afford lowest-cost nutritious diets in the optimal scenario of combined access to multiple interventions.

In conclusion, our results have several important policy implications. Behavior change communication strategies should focus on raising community awareness through tailored messages applicable to the local context to improve existing dietary patterns, particularly among pregnant women. Examples of such strategies could include counseling on specific nutrient-rich foods that are affordable and locally available in different seasons and how they can be combined with the commodities available through the public-sector programs. On the supply side, our results suggest that scaling-up and intensifying home production and social protection strategies could enable more households to obtain nutritious diets. Policy measures toward this could include promotion of kitchen gardens for diet diversification, improving delivery mechanisms and quality of food transfers (PDS and THRs), and strengthening the financial infrastructure for cash transfers. Behavior change communication interventions should link with other programs that can together help find ways to bridge the nutrient gaps with a combination of home production and commodities available in the markets. Although our results provide indications of the extent to which individual and combined interventions could improve affordability, future research on the feasibility and effectiveness of specific interventions in different contexts is recommended.

## Supplementary Material

nzaa169_Supplemental_File

## Notes

This work was supported by the Bill & Melinda Gates Foundation, Seattle, WA (grant number OPP1150189).

Author disclosures: The authors report no conflicts of interest.

Supplemental Tables 1–7 are available from the “Supplementary data” link in the online posting of the article and from the same link in the online table of contents at https://academic.oup.com/cdn/.

Abbreviations used: A&T, Alive and Thrive; CotD, Cost of the Diet; CPI, Consumer Price Index; GP, Gram Panchayat; INR, Indian rupees; PDS, Public Distribution System; THR, take-home ration; US$, US dollars.
