# Supplementary material for: Assessing the Economic Feasibility of Assuring Nutritionally Adequate Diets for Vulnerable Populations in Uttar Pradesh, India: Findings from a “Cost of the Diet” Analysis
Source: Curr Dev Nutr. 2020 Nov 13;4(12):nzaa169. doi: 10.1093/cdn/nzaa169 (PMC7721462; doi:10.1093/cdn/nzaa169)
Supplement: nzaa169_Supplemental_File [file nzaa169_supplemental_file.docx]

**Supplemental table 1: Cost, consumption, and availability of 137 food items**

| **Food item (local name)** | **Price per**  **100 g (INR)** | **Food frequency score** | **Availability (out of 24 markets)** |
| --- | --- | --- | --- |
| **Grains, white roots and tubers, and plantains** | | | |
| White bread | 6.54 | 9.18 | 23 |
| Maize | 3.37 | 6.82 | 9 |
| Rice flaked | 4.34 | 7.04 | 24 |
| Rice paddy | 2.74 | 14.30 | 20 |
| Rice puffed | 5.22 | 6.53 | 22 |
| Vermicelli | 6.72 | 5.15 | 21 |
| Wheat flour | 2.39 | 16.00 | 17 |
| Refined wheat flour | 2.52 | 4.26 | 23 |
| Pearl millet (Bajra) | 3.79 | 4.06 | 2 |
| Cracked wheat (Dalia) | 4.78 | 6.82 | 10 |
| Tapioca (Saboodana) | 6.97 | 3.78 | 21 |
| Semolina (Sooji) | 2.74 | 4.70 | 24 |
| Potato | 1.84 | 14.34 | 24 |
| Sweet potato | 2.71 | 6.62 | 3 |
| Turnip | 2.25 | 2.18 | 6 |
| Colocasia (Arbi) | 2.67 | 6.14 | 19 |
| Lotus stem | 3.39 | 2.43 | 1 |
| Plantain | 1.82 | 3.42 | 15 |
| **Pulses (beans, peas, and lentils)** | | | |
| Lentil dried (Arhar) | 6.29 | 10.43 | 23 |
| Chickpea flour (Besan) | 7.18 | 7.07 | 23 |
| Bengal gram split | 6.11 | 6.98 | 24 |
| Bengal gram whole | 5.35 | 5.98 | 24 |
| White chickpea | 7.62 | 4.48 | 22 |
| Cowpea (Lobia) | 2.97 | 6.62 | 11 |
| Pink lentil (Masoor dal) | 5.98 | 3.14 | 24 |
| Lentil whole (Masoor) | 5.63 | 5.25 | 20 |
| Green gram split | 6.68 | 6.11 | 21 |
| Green gram whole | 5.75 | 1.76 | 15 |
| Kidney bean | 8.68 | 1.98 | 17 |
| Soya bean | 7.84 | 7.17 | 23 |
| Black gram split | 6.21 | 7.36 | 23 |
| Black gram whole | 4.61 | 1.22 | 14 |
| **Nuts and seeds** | | | |
| Almond | 78.7 | 5.12 | 19 |
| Cashew nut | 89.23 | 3.74 | 17 |
| Peanut | 8.57 | 9.47 | 16 |
| Pistachio | 138.65 | 0.64 | 9 |
| Walnut | 112.43 | 0.70 | 6 |
| Lotus seeds | 65.22 | 4.13 | 23 |
| **Dairy** | | | |
| Buttermilk | 3.2 | 8.19 | 6 |
| Cottage cheese | 21.56 | 4.26 | 17 |
| Buffalo milk | 3.93 | 11.65 | 24 |
| Cow milk | 3.77 | 5.82 | 24 |
| Yoghurt | 8.99 | 9.18 | 17 |
| Evaporated cow milk | 19.71 | 5.79 | 10 |
| **Meat, poultry, and fish** | | | |
| Chicken | 14.13 | 3.26 | 23 |
| Chicken liver | 9.57 | 1.44 | 3 |
| Goat | 31.05 | 1.92 | 15 |
| Goat intestines | 6.44 | 0.54 | 10 |
| Goat feet | 18.11 | 0.67 | 5 |
| Chicken breast | 8.0 | 1.06 | 1 |
| Chicken leg | 2.86 | 1.70 | 1 |
| Pangas fish | 11.55 | 0.64 | 11 |
| Bacha fish (Bachua) | 10.78 | 0.38 | 7 |
| Minnow fish (Chilwa) | 5.56 | 0.99 | 7 |
| Katla fish (Catla) | 10.58 | 0.48 | 3 |
| Catfish (Mangur) | 15.27 | 1.79 | 10 |
| Other fish | 9.55 | 1.47 | 12 |
| Boal fish (Pardni) | 17.57 | 1.22 | 15 |
| Rohu fish | 12.92 | 1.60 | 9 |
| Tenger fish (Tengra) | 14.6 | 1.22 | 5 |
| **Eggs** | | | |
| Chicken egg | 10.62 | 4.06 | 24 |
| **Dark green leafy vegetables** | | | |
| Cabbage | 1.76 | 8.51 | 24 |
| Red amaranth leaves | 1.47 | 1.22 | 1 |
| Chickpea leaf | 4.74 | 6.27 | 1 |
| Mustard spinach | 1.79 | 6.40 | 11 |
| Turnip leaf | 3.04 | 0.70 | 2 |
| Radish leaf | 1.02 | 9.09 | 23 |
| Spinach | 1.53 | 8.10 | 24 |
| Colocasia leaf | 2.54 | 3.14 | 2 |
| Goosefoot leaf (Bathua) | 5.81 | 8.51 | 7 |
| Fenugreek leaf (Methi) | 4.05 | 8.10 | 92 |
| Soya leaves | 3.79 | 6.88 | 22 |
| **Other Vitamin-A rich fruits and vegetables** | | | |
| Carrot | 3.99 | 8.03 | 13 |
| Pumpkin | 1.35 | 7.01 | 18 |
| Papaya | 3.65 | 5.98 | 9 |
| **Other vegetables** | | | |
| Cluster bean | 1.85 | 6.24 | 20 |
| French bean | 5.26 | 1.31 | 4 |
| Brinjal | 1.43 | 7.46 | 24 |
| Cauliflower | 1.65 | 9.06 | 24 |
| Cucumber | 3.02 | 9.28 | 16 |
| Okra | 1.75 | 8.45 | 23 |
| Green onion | 3.2 | 5.18 | 14 |
| Onion | 2.17 | 14.72 | 24 |
| Peas | 9.74 | 9.63 | 18 |
| Water chestnut | 1.77 | 8.45 | 23 |
| Bitter gourd (Karela) | 2.15 | 7.17 | 23 |
| Bottle gourd (Lauki) | 1.07 | 7.78 | 23 |
| Cowpea pod | 2.58 | 6.18 | 19 |
| Pointed gourd (Parwal) | 2.91 | 7.87 | 24 |
| Ash gourd (Petha) | 2.23 | 2.56 | 5 |
| Broad beans (Sem) | 5.75 | 7.33 | 11 |
| Green pepper | 3.61 | 5.50 | 24 |
| Ridge gourd (Tori) | 1.92 | 8.48 | 20 |
| Tomato | 2.22 | 14.50 | 24 |
| Beetroot | 4.47 | 4.77 | 12 |
| **Other fruits** | | | |
| Apple | 5.72 | 7.94 | 24 |
| Banana | 2.38 | 8.35 | 22 |
| Coconut | 19.61 | 4.83 | 15 |
| Dates | 19.66 | 2.24 | 7 |
| Fig | 99.64 | 0.03 | 2 |
| Grapes | 18.19 | 7.17 | 7 |
| Guava | 2.71 | 9.18 | 7 |
| Orange | 4.89 | 6.18 | 11 |
| Peach | 3.53 | 0.42 | 1 |
| Pineapple | 4.0 | 2.37 | 11 |
| Pomegranate | 5.6 | 7.78 | 21 |
| Raisin | 29.67 | 4.32 | 19 |
| Tamarind | 11.27 | 2.88 | 11 |
| Emblic (Amla) | 2.04 | 1.54 | 4 |
| Custard apple (Sitaphal) | 6.55 | 2.46 | 8 |
| Sweet lime | 3.69 | 4.16 | 13 |
| Lemon | 9.55 | 8.67 | 23 |
| **Other oils and fats** | | | |
| Ghee | 48.82 | 8.99 | 16 |
| Mustard oil | 9.95 | 15.62 | 23 |
| Sunflower oil | 10.99 | 0.22 | 1 |
| Vegetable oil | 8.06 | 1.92 | 12 |
| Soybean oil | 9.41 | 2.21 | 22 |
| **Sweets and snacks** |  |  |  |
| Biscuit (sweet) | 9.22 | 10.08 | 24 |
| Biscuit (salty) | 10.19 | 10.02 | 24 |
| Jaggery | 4.55 | 7.78 | 21 |
| White sugar | 3.74 | 15.39 | 24 |
| **Condiments and beverages** | | | |
| Betel leaf | 42.67 | 2.05 | 21 |
| Green chili | 4.83 | 14.27 | 24 |
| Red chili powder | 24.5 | 13.41 | 24 |
| Coriander powder | 14.21 | 14.59 | 24 |
| Coriander leaf | 6.42 | 13.38 | 24 |
| Cumin seeds | 24.92 | 13.63 | 24 |
| Fenugreek seeds | 7.86 | 7.33 | 22 |
| Garlic | 3.26 | 14.69 | 24 |
| Ginger | 9.56 | 13.76 | 24 |
| Mint leaf | 10.09 | 2.88 | 7 |
| Mustard seeds | 4.79 | 3.36 | 11 |
| Black pepper | 60.51 | 11.10 | 24 |
| Salt | 1.24 | 16.00 | 24 |
| Turmeric | 16.99 | 15.74 | 24 |
| Caraway seeds (Ajwain) | 20.69 | 5.73 | 21 |
| Tea powder | 17.1 | 15.20 | 24 |

**Supplemental table 2: Nutrient requirements comparison between WHO/FAO and NIN RDA**

| **Nutrient** | **WHO/FAO** | | | | | | | **NIN RDA** | | | | |
| --- | --- | --- | --- | --- | --- | --- | --- | --- | --- | --- | --- | --- |
|  | **Child**  **(12-23m)** | **Child**  **(3-4y)** | **Child**  **(5-6y)** | **Man,**  **medium activity** | **Woman, light activity** | **Woman, medium activity, pregnant** | **Child**  **(1-3y)** | | **Child**  **(4-6y)** | **Man,**  **medium activity** | **Woman,**  **light activity** | **Woman medium activity, pregnant** |
| Energy (kcal) | 906.5 | 1204 | 1398.5 | 3050 | 1800 | 2732 | 1060 | | 1350 | 2730 | 1900 | 2580 |
| Protein (g) | 13.55 | 13.76 | 16.01 | 48.48 | 40.4 | 57.85 | 16.7 | | 20.1 | 60 | 55 | 78 |
| Fat (g) | 30.2 | 33.44 | 38.85 | 67.78 | 40 | 60.71 | 27 | | 25 | 30 | 25 | 30 |
| Vitamin A (ug retinol equivalent) | 400 | 400 | 450 | 600 | 600 | 800 | 400 | | 400 | 600 | 600 | 800 |
| Vitamin C (mg) | 30 | 30 | 30 | 45 | 45 | 55 | 40 | | 40 | 40 | 40 | 60 |
| Vitamin B1 (mg) | 0.5 | 0.5 | 0.6 | 1.2 | 1.1 | 1.4 | 0.5 | | 0.7 | 1.4 | 1 | 1.3 |
| Vitamin B2 (mg) | 0.5 | 0.5 | 0.6 | 1.3 | 1.1 | 1.4 | 0.6 | | 0.8 | 1.6 | 1.1 | 1.6 |
| Niacin (mg) | 6 | 6 | 8 | 16 | 14 | 18 | 8 | | 11 | 18 | 12 | 16 |
| Vitamin B6 (mg) | 0.5 | 0.5 | 0.6 | 1.3 | 1.5 | 1.9 | 0.9 | | 0.9 | 2 | 2 | 2.5 |
| Pantothenic Acid (mg) | 2 | 2 | 3 | 5 | 5 | 6 | - | | - | - | - | - |
| Folic Acid (ug DFE) | 150 | 150 | 200 | 400 | 400 | 600 | 80 | | 100 | 200 | 200 | 500 |
| Vitamin B12 (ug) | 0.9 | 0.9 | 1.2 | 2.4 | 2.4 | 2.6 | 0.2-1 | | 0.2-1 | 1 | 1 | 1.2 |
| Calcium (mg) | 500 | 500 | 600 | 1000 | 1300 | 1060 | 600 | | 600 | 600 | 600 | 1200 |
| Iron (mg) | 0.58 | 0.58 | 0.63 | 1.37 | 1.13 | 2.94 | 9 | | 13 | 17 | 21 | 35 |
| Magnesium (mg) | 60 | 60 | 76 | 260 | 190 | 220 | 50 | | 70 | 340 | 310 | 310 |
| Zinc (mg) | 4.1 | 4.1 | 4.8 | 7 | 4.9 | 7.45 | 5 | | 7 | 12 | 10 | 12 |

**Supplemental table 3: Nutrient composition per 100g of take-home ration (THR) entered in the software**

| Energy (kcal) | 335.47 |
| --- | --- |
| Protein (g) | 12.23 |
| Fat (g) | 9.31 |
| Carbohydrate (g) | 57.49 |
| Fibre (g) | 8.87 |
| Retinol (μg) | 0.00 |
| Vitamin C (mg) | 0.00 |
| Vitamin B1 (mg) | 0.29 |
| Vitamin B2 (mg) | 0.10 |
| Niacin (mg) | 1.56 |
| Vitamin B6 (mg) | 0.38 |
| Folate (µg) | 67.78 |
| Vitamin B12 (mg) | 0.00 |
| Pantothenic acid (mg) | 0.80 |
| Calcium (mg) | 73.72 |
| Iron (mg) | 4.23 |
| Magnesium (mg) | 114.84 |
| Zinc (mg) | 1.99 |

**Supplemental table 4: Annual composition of energy only diet**

| **Food list** | **Quantity**  **(kg)** | **% quantity** | **Cost (INR)** | **%**  **cost** | **% energy** | **% protein** | **%**  **fat** | **%**  **vit A** | **%**  **vit C** | **%**  **vit B1** | **%**  **vit B2** | **% niacin** | **%**  **vit B6** | **%**  **folic acid** | **%**  **vit B12** | **% calcium** | **%**  **iron** | **%**  **zinc** |
| --- | --- | --- | --- | --- | --- | --- | --- | --- | --- | --- | --- | --- | --- | --- | --- | --- | --- | --- |
| Breast milk | 194 | 14.5 | 0 | 0 | 3.1 | 1.3 | 27.3 | 100.0 | 100.0 | 1.1 | 3.4 | 0.9 | 0.6 | 4.7 | 100.0 | 13.2 | 0.0 | 0.8 |
| Whole wheat flour | 739 | 55.2 | 17663 | 63.3 | 61.9 | 66.1 | 50.7 | 0.0 | 0.0 | 70.8 | 70.2 | 70.7 | 72.5 | 65.2 | 0.0 | 61.1 | 71.7 | 72.0 |
| Wheat flour | 406 | 30.3 | 10229 | 36.7 | 35.0 | 32.6 | 22.0 | 0.0 | 0.0 | 28.1 | 26.4 | 28.4 | 27.0 | 30.1 | 0.0 | 25.7 | 28.3 | 27.3 |
| Total | 1339 | 100 | 27892 | 100 | 100 | 100 | 100 | 100 | 100 | 100 | 100 | 100 | 100 | 100 | 100 | 100 | 100 | 100 |
| % target met |  | | | | 100 | 221 | 28 | 8 | 9 | 194 | 101 | 389 | 137 | 51 | 5 | 23 | 76 | 252 |

**Supplemental table 5: Annual composition of macronutrient diet**

| **Food list** | **Quantity**  **(kg)** | **% quantity** | **Cost (INR)** | **% cost** | **% energy** | **% protein** | **% fat** | **%**  **vit A** | **%**  **vit C** | **%**  **vit B1** | **%**  **vit B2** | **% niacin** | **%**  **vit B6** | **%**  **folic acid** | **%**  **vit B12** | **% calcium** | **% iron** | **%**  **zinc** |
| --- | --- | --- | --- | --- | --- | --- | --- | --- | --- | --- | --- | --- | --- | --- | --- | --- | --- | --- |
| Breast milk | 194 | 15.8 | 0 | 0.0 | 3.1 | 1.6 | 7.7 | 100.0 | 100.0 | 1.2 | 3.9 | 1.0 | 0.7 | 5.4 | 100.0 | 15.0 | 0.0 | 0.9 |
| Vegetable oil | 74 | 6.0 | 5963 | 20.4 | 15.8 | 0.0 | 74.8 | 0.0 | 0.0 | 0.0 | 0.0 | 0.0 | 0.0 | 0.0 | 0.0 | 0.0 | 0.0 | 0.0 |
| Whole wheat flour | 733 | 59.6 | 17516 | 59.9 | 61.4 | 76.9 | 14.1 | 0.0 | 0.0 | 80.6 | 79.2 | 80.5 | 82.0 | 74.9 | 0.0 | 68.6 | 81.7 | 81.5 |
| Wheat flour | 229 | 18.6 | 5775 | 19.7 | 19.8 | 21.6 | 3.5 | 0.0 | 0.0 | 18.2 | 16.9 | 18.5 | 17.4 | 19.6 | 0.0 | 16.4 | 18.3 | 17.6 |
| Total | 1230 | 100 | 29254 | 100 | 100 | 100 | 100 | 100 | 100 | 100 | 100 | 100 | 100 | 100 | 100 | 100 | 100 | 100 |
| % target met |  | | | | 100 | 188 | 100 | 8 | 9 | 169 | 89 | 339 | 121 | 44 | 5 | 20 | 66 | 221 |

**Supplemental table 6: Annual composition of nutritious diet**

| **Food list** | **Quantity**  **(kg)** | **% quantity** | **Cost (INR)** | **% cost** | **% energy** | **% protein** | **% fat** | **%**  **vit A** | **%**  **vit C** | **%**  **vit B1** | **%**  **vit B2** | **% niacin** | **%**  **vit B6** | **%**  **folic acid** | **%**  **vit B12** | **% calcium** | **% iron** | **% zinc** |
| --- | --- | --- | --- | --- | --- | --- | --- | --- | --- | --- | --- | --- | --- | --- | --- | --- | --- | --- |
| Pearl millet | 33 | 2.0 | 1257 | 2.9 | 3.0 | 2.5 | 1.7 | 0.1 | 0.0 | 2.6 | 2.8 | 2.1 | 2.6 | 1.3 | 0.0 | 0.8 | 3.4 | 3.3 |
| Breast milk | 194 | 11.6 | 0 | 0.0 | 3.1 | 1.3 | 7.7 | 2.3 | 4.0 | 1.2 | 2.5 | 1.0 | 0.5 | 2.0 | 4.5 | 3.0 | 0.0 | 0.8 |
| Chicken liver | 20 | 1.2 | 1921 | 4.5 | 0.6 | 2.2 | 1.0 | 15.4 | 0.0 | 1.9 | 13.2 | 2.1 | 4.6 | 14.3 | 79.8 | 0.2 | 11.6 | 1.6 |
| Minnow fish | 19 | 1.2 | 1495 | 3.5 | 0.6 | 2.3 | 1.0 | 0.0 | 0.0 | 0.6 | 0.8 | 0.0 | 1.7 | 0.0 | 15.7 | 3.8 | 6.7 | 2.0 |
| Jaggery | 54 | 3.2 | 2451 | 5.7 | 4.7 | 0.5 | 0.2 | 0.0 | 0.0 | 0.0 | 0.0 | 0.0 | 0.0 | 0.0 | 0.0 | 10.8 | 0.0 | 0.0 |
| Amaranth leaf | 444 | 26.6 | 9452 | 21.9 | 3.5 | 13.2 | 1.5 | 82.1 | 95.7 | 4.0 | 21.4 | 8.3 | 23.0 | 45.7 | 0.0 | 62.7 | 34.2 | 13.8 |
| Mustard seeds | 15 | 0.9 | 713 | 1.7 | 2.0 | 2.4 | 6.9 | 0.1 | 0.2 | 1.0 | 1.8 | 1.5 | 0.9 | 1.0 | 0.0 | 0.4 | 2.9 | 3.6 |
| Vegetable oil | 64 | 3.8 | 5153 | 11.9 | 13.6 | 0.0 | 64.6 | 0.0 | 0.0 | 0.0 | 0.0 | 0.0 | 0.0 | 0.0 | 0.0 | 0.0 | 0.0 | 0.0 |
| Okra | 2 | 0.1 | 46 | 0.1 | 0.0 | 0.0 | 0.0 | 0.0 | 0.2 | 0.0 | 0.1 | 0.0 | 0.1 | 0.2 | 0.0 | 0.1 | 0.0 | 0.0 |
| Black gram | 38 | 2.3 | 1765 | 4.1 | 3.0 | 5.6 | 0.6 | 0.0 | 0.0 | 4.9 | 3.7 | 2.6 | 2.9 | 6.5 | 0.0 | 3.9 | 2.9 | 2.9 |
| Whole wheat flour | 712 | 42.6 | 17011 | 39.4 | 59.6 | 63.9 | 13.7 | 0.0 | 0.0 | 78.0 | 50.1 | 76.5 | 59.6 | 26.7 | 0.0 | 13.4 | 35.6 | 67.2 |
| Wheat flour | 74 | 4.4 | 1862 | 4.3 | 6.4 | 6.0 | 1.1 | 0.0 | 0.0 | 5.8 | 3.6 | 5.8 | 4.2 | 2.3 | 0.0 | 1.1 | 2.7 | 4.8 |
| Total | 1669 | 100 | 43128 | 100 | 100 | 100 | 100 | 100 | 100 | 100 | 100 | 100 | 100 | 100 | 100 | 100 | 100 | 100 |
| % target met |  | | | | 100 | 220 | 100 | 361 | 227 | 170 | 137 | 346 | 161 | 119 | 111 | 100 | 148 | 260 |

**Supplemental table 7: Annual composition of food habits-based nutritious diet**

| **Food list** | **Quantity**  **(kg)** | **% quantity** | **Cost (INR)** | **%**  **cost** | **% energy** | **% protein** | **%**  **fat** | **%**  **vit A** | **%**  **vit C** | **%**  **vit B1** | **%**  **vit B2** | **% niacin** | **%**  **vit B6** | **%**  **folic acid** | **%**  **vit B12** | **% calcium** | **%**  **iron** | **% zinc** |
| --- | --- | --- | --- | --- | --- | --- | --- | --- | --- | --- | --- | --- | --- | --- | --- | --- | --- | --- |
| Colocasia leaf | 27 | 1.3 | 859 | 1.3 | 0.3 | 0.7 | 0.3 | 9.3 | 14.9 | 1.7 | 1.9 | 0.9 | 1.2 | 3.9 | 0.0 | 3.3 | 2.5 | 0.6 |
| Pearl millet | 99 | 4.8 | 3738 | 5.8 | 8.8 | 7.0 | 4.9 | 0.6 | 0.0 | 7.2 | 6.0 | 7.0 | 8.2 | 3.6 | 0.0 | 2.2 | 14.9 | 10.2 |
| Chickpea flour | 4 | 0.2 | 297 | 0.5 | 0.5 | 0.7 | 0.3 | 0.0 | 0.1 | 0.4 | 0.2 | 0.3 | 0.5 | 2.6 | 0.0 | 0.3 | 0.7 | 0.7 |
| Breast milk | 194 | 9.4 | 0 | 0.0 | 3.1 | 1.3 | 7.5 | 5.6 | 8.9 | 1.1 | 1.8 | 1.1 | 0.5 | 1.9 | 5.0 | 2.8 | 0.0 | 0.8 |
| Buttermilk | 31 | 1.5 | 1006 | 1.6 | 0.3 | 0.7 | 0.1 | 0.0 | 0.4 | 0.4 | 1.2 | 0.4 | 0.4 | 0.2 | 3.1 | 2.0 | 0.0 | 0.4 |
| Bengal gram | 22 | 1.1 | 1197 | 1.9 | 1.9 | 2.8 | 1.3 | 0.0 | 0.0 | 1.9 | 1.6 | 1.4 | 3.4 | 4.8 | 0.0 | 2.4 | 3.7 | 2.0 |
| Egg | 45 | 2.2 | 5285 | 8.2 | 1.7 | 3.5 | 4.7 | 4.9 | 0.0 | 0.9 | 6.0 | 1.5 | 1.5 | 2.3 | 13.1 | 1.2 | 5.1 | 1.7 |
| Fenugreek seeds | 2 | 0.1 | 120 | 0.2 | 0.1 | 0.2 | 0.1 | 0.0 | 0.0 | 0.1 | 0.1 | 0.1 | 0.3 | 0.1 | 0.0 | 0.1 | 0.7 | 0.2 |
| Guava | 4 | 0.2 | 113 | 0.2 | 0.1 | 0.0 | 0.0 | 0.1 | 11.0 | 0.2 | 0.1 | 0.1 | 0.1 | 0.2 | 0.0 | 0.0 | 0.1 | 0.0 |
| Jaggery | 9 | 0.4 | 409 | 0.6 | 0.8 | 0.1 | 0.0 | 0.0 | 0.0 | 0.0 | 0.0 | 0.0 | 0.0 | 0.0 | 0.0 | 1.7 | 0.0 | 0.0 |
| Mustard spinach | 20 | 1.0 | 467 | 0.7 | 0.1 | 0.3 | 0.1 | 5.7 | 29.6 | 0.4 | 0.5 | 2.0 | 0.9 | 3.6 | 0.0 | 2.2 | 0.6 | 0.1 |
| Buffalo milk | 650 | 31.6 | 25554 | 39.8 | 15.9 | 16.3 | 43.1 | 23.0 | 7.5 | 9.2 | 29.2 | 8.9 | 9.3 | 4.5 | 78.8 | 50.7 | 0.0 | 10.9 |
| Mustard seeds | 5 | 0.2 | 238 | 0.4 | 0.7 | 0.7 | 2.3 | 0.1 | 0.1 | 0.3 | 0.4 | 0.6 | 0.3 | 0.3 | 0.0 | 0.1 | 1.4 | 1.2 |
| Soya bean oil | 17 | 0.8 | 1597 | 2.5 | 3.7 | 0.0 | 16.8 | 0.0 | 0.0 | 0.0 | 0.0 | 0.0 | 0.0 | 0.0 | 0.0 | 0.0 | 0.0 | 0.0 |
| Salt | 2 | 0.1 | 31 | 0.0 | 0.0 | 0.0 | 0.0 | 0.0 | 0.0 | 0.0 | 0.0 | 0.0 | 0.0 | 0.0 | 0.0 | 0.0 | 0.0 | 0.0 |
| Semolina | 21 | 1.0 | 581 | 0.9 | 1.8 | 1.4 | 0.3 | 0.0 | 0.0 | 1.1 | 0.3 | 1.1 | 0.5 | 1.4 | 0.0 | 0.2 | 0.4 | 1.5 |
| Soya bean | 29 | 1.4 | 2299 | 3.6 | 3.2 | 6.6 | 5.8 | 0.0 | 2.0 | 7.2 | 6.7 | 0.6 | 3.2 | 12.6 | 0.0 | 4.3 | 8.7 | 4.8 |
| Soya leaves | 1 | 0.1 | 43 | 0.1 | 0.0 | 0.0 | 0.0 | 0.3 | 1.1 | 0.0 | 0.1 | 0.0 | 0.1 | 0.2 | 0.0 | 0.1 | 0.1 | 0.0 |
| Spinach | 213 | 10.3 | 4068 | 6.3 | 1.2 | 3.9 | 0.6 | 50.4 | 24.4 | 6.0 | 13.5 | 3.2 | 14.7 | 35.5 | 0.0 | 15.1 | 14.5 | 5.7 |
| Turmeric | 2 | 0.1 | 384 | 0.6 | 0.2 | 0.1 | 0.2 | 0.0 | 0.0 | 0.1 | 0.1 | 0.1 | 1.2 | 0.1 | 0.0 | 0.2 | 1.4 | 0.3 |
| Whole wheat flour | 486 | 23.7 | 11622 | 18.1 | 40.7 | 40.8 | 9.1 | 0.0 | 0.0 | 49.3 | 24.4 | 57.7 | 43.3 | 17.2 | 0.0 | 8.7 | 35.9 | 47.2 |
| Wheat flour | 171 | 8.3 | 4316 | 6.7 | 14.8 | 12.9 | 2.5 | 0.0 | 0.0 | 12.5 | 5.9 | 14.9 | 10.3 | 5.1 | 0.0 | 2.3 | 9.1 | 11.5 |
| Total | 2056 | 100 | 64225 | 100 | 100 | 100 | 100 | 100 | 100 | 100 | 100 | 100 | 100 | 100 | 100 | 100 | 100 | 100 |
| % target met |  | | | | 100 | 235 | 102 | 146 | 102 | 184 | 192 | 314 | 151 | 126 | 100 | 106 | 100 | 253 |
